# Retraction Note: FXR activation alleviates tacrolimus-induced post-transplant diabetes mellitus by regulating renal gluconeogenesis and glucose uptake

**DOI:** 10.1186/s12967-026-07893-w

**Published:** 2026-02-19

**Authors:** Ling Li, Huijia Zhao, Binyao Chen, Zhipeng Fan, Ning Li, Jiang Yue, Qifa Ye

**Affiliations:** 1 Zhongnan Hospital of Wuhan University, Institute of Hepatobiliary Diseases of Wuhan University, Transplant Center of Wuhan University, Hubei Key Laboratory of Medical Technology on Transplantation, Wuhan, 430071 People’s Republic of China; 2 The 3rd Xiangya Hospital of Central South University, Research Center of National Health Ministry on Transplantation Medicine Engineering and Technology, Changsha, 410013 People’s Republic of China; 3 Department of Pharmacology, Basic Medical School of Wuhan University, Wuhan, 430071 People’s Republic of China; 4 Department of Cardiology, Renmin Hospital of Wuhan University, Cardiovascular Research Institute, Hubei Key Laboratory of Cardiology, Wuhan University, Jiefang Road 238, Wuhan, 430060 People’s Republic of China

**Journal of Translational Medicine (2019) 17:418**

10.1186/s12967-019-02170-5

The Editor has retracted this article. Initially, the authors alerted the journal that the image corresponding to the FK506+GW4064 group in Fig. 1e was inadvertently reused due to an operational oversight. However, a further investigation found further issues, such as the same image being used in Fig 1d, Fig 1f, and Fig 2a, where it is marked differently (Histone 3 and GADPH). The authors failed to provide raw unchanged images on request. The Editor has therefore lost confidence in the findings reported in this article. The authors agree to the retraction but not to the retraction wording.

